# Molecular Genetics of Niemann–Pick Type C Disease in Italy: An Update on 105 Patients and Description of 18 *NPC1* Novel Variants

**DOI:** 10.3390/jcm9030679

**Published:** 2020-03-03

**Authors:** Andrea Dardis, Stefania Zampieri, Cinzia Gellera, Rosalba Carrozzo, Silvia Cattarossi, Paolo Peruzzo, Rosalia Dariol, Annalisa Sechi, Federica Deodato, Claudio Caccia, Daniela Verrigni, Serena Gasperini, Agata Fiumara, Simona Fecarotta, Miryam Carecchio, Massimiliano Filosto, Lucia Santoro, Barbara Borroni, Andrea Bordugo, Francesco Brancati, Cinzia V. Russo, Maja Di Rocco, Antonio Toscano, Maurizio Scarpa, Bruno Bembi

**Affiliations:** 1 Regional Coordinator Centre for Rare Diseases, University Hospital of Udine, 33100 Udine, Italy; stefania.zampieri@asufc.sanita.fvg.it (S.Z.); silvia.cattarossi@asufc.sanita.fvg.it (S.C.); paolo.peruzzo@asufc.sanita.fvg.it (P.P.); rosalia.dariol@asufc.sanita.fvg.it (R.D.); annalisa.sechi@asufc.sanita.fvg.it (A.S.); maurizio.scarpa@asufc.sanita.fvg.it (M.S.); brunobembi.b@gmail.com (B.B.); 2 Unit of Genetics of Neurodegenerative and Metabolic Diseases Fondazione IRCCS Istituto Neurologico Carlo Besta, 20133 Milan, Italy; Cinzia.Gellera@istituto-besta.it (C.G.); Claudio.Caccia@istituto-besta.it (C.C.); mcarecchio@gmail.com (M.C.); 3 Unit of Muscular and Neurodegenerative Disorders, Laboratory of Molecular Medicine, Bambino Gesù Children’s Hospital, IRCCS, 00146 Rome, Italy; rosalba.carrozzo@opbg.net (R.C.); sambadan@hotmail.com (D.V.); 4 Division of Metabolism, Departement of Pediatric Subspecialities, Bambino Gesù Children’s Hospital, IRCCS, 00146 Rome, Italy; federica.deodato@opbg.net; 5 Pediatric Rare Diseases Unit, Department of Pediatrics, MBBM Foundation, ATS Monza e Brianza, 20900 Monza, Italy; serena.gasperini69@gmail.com; 6 Department of Pediatrics, Regional Referral Center for Inherited Metabolic Disease, University of Catania, 95123 Catania, Italy; agatafiumara@yahoo.it; 7 Department of Pediatrics “Federico II” University Hospital, 80131 Naples, Italy; simona.fecarotta@unina.it; 8 Department of Neuroscience, University of Padua, Via Giustiniani 2, 35128 Padua, Italy; 9 Center for Neuromuscular Diseases, Unit of Neurology, ASST “Spedali Civili” and University of Brescia, 25123 Brescia, Italy; massimiliano.filosto@unibs.it; 10 Department of Clinical Sciences, Division of Pediatrics, Polytechnic University of Marche, Ospedali Riuniti, 60020 Ancona, Italy; dott.luciasantoro@gmail.com; 11 Centre for Neurodegenerative Disorders, Department of Clinical and Experimental Sciences, University of Brescia, 25123 Brescia, Italy; bborroni@inwind.it; 12 Inherited Metabolic Disease Unit and Regional Centre for Newborn Screening, Children and Women Hospital, Azienda Ospedaliera Università Integrata, 37126 Verona, Italy; andrea.bordugo@aovr.veneto.it; 13 Medical Genetics Unit, Tor Vergata University, 00133 Roma, Italy; geneticaclinica@ptvonline.it; 14 Department of Neurosciences, Reproductive and Odontostomatological Sciences, Federico II University, 80138 Naples, Italy; cinziavaleria@hotmail.it; 15 Department of Pediatrics, Unit of Rare Diseases, Giannina Gaslini Institute, 16147 Genoa, Italy; majadirocco@gaslini.org; 16 Department of Neurosciences, University of Messina, 98125 Messina, Italy; atoscano@unime.it

**Keywords:** Niemann–Pick C disease, NPC1, NPC2, mutations

## Abstract

Niemann-Pick type C (NPC) disease is an autosomal recessive lysosomal storage disorder caused by mutations in *NPC1* or *NPC2* genes. In 2009, the molecular characterization of 44 NPC Italian patients has been published. Here, we present an update of the genetic findings in 105 Italian NPC patients belonging to 83 unrelated families (77 NPC1 and 6 NPC2). *NPC1* and *NPC2* genes were studied following an algorithm recently published. Eighty-four different *NPC1* and five *NPC2* alleles were identified. Only two *NPC1* alleles remained non detected. Sixty-two percent of *NPC1* alleles were due to missense variants. The most frequent *NPC1* mutation was the p.F284Lfs*26 (5.8% of the alleles). All *NPC2* mutations were found in the homozygous state, and all but one was severe. Among newly diagnosed patients, 18 novel *NPC1* mutations were identified. The pathogenic nature of 7/9 missense alleles and 3/4 intronic variants was confirmed by filipin staining and NPC1 protein analysis or mRNA expression in patient’s fibroblasts. Taken together, our previous published data and new results provide an overall picture of the molecular characteristics of NPC patients diagnosed so far in Italy.

## 1. Introduction

Niemann–Pick type C disease (NPC-MIM 257220; MIM607625) is an autosomal recessive lysosomal storage disorder due to mutations in *NPC1* (95% of patients) or *NPC2* genes, encoding two proteins involved in the intracellular trafficking of cholesterol and other lipids. The deficiency of either protein leads to the accumulation of endocytosed unesterified cholesterol, gangliosides, and other lipids within the lysosome/late endosome compartment [1]. The incidence of NPC is estimated to be one in every 100,000 live births [1]. However, the evaluation of common *NPC1* variants suggests that the late-onset NPC1 phenotype may have a much higher incidence, ranging from 1:19,000 to 1:36,000 [2].

The clinical presentation of the disease is variable, and the age at onset ranges from the perinatal period to adulthood. The disease is typically characterized by visceral and neurological symptoms. Apart from a small group of patients presenting with a severe perinatal form leading to death within the first year of life due to liver or respiratory failure, most patients develop a progressive neurological disease. Indeed, NPC has been traditionally classified on the basis of the age at onset of the neurological symptoms, irrespective of the age of first symptom [1].

*NPC1* (MIM 607623; chr 18q11-q12) gene encodes a membrane glycoprotein of 1278 amino acids containing 3 luminal domains: N-terminal domain (NTD), middle luminal domain (MLD), C-terminal domain (CTD, also called cysteine-rich domain, and 13 transmembrane helices (TMs) [3]. The TM3-7 segment, termed sterol-sensing domain (SSD), is highly conserved within a subclass of membrane proteins in the cholesterol metabolism pathway [4,5]. *NPC2* (MIM 601015; chr 14q24.3) encodes a soluble 151 aminoacid protein present in the lumen of lysosomes [1].

NPC1 and NPC2 proteins participate together and sequentially in mediating the egress of unesterified cholesterol from the endo/lysosomal compartment. The precise mechanism by which these proteins function is not fully understood. However, based on recently published data, a hydrophobic hand-off cholesterol transfer model has been hypothesized [6]. According to this model, cholesterol binding to the NPC2 in the lumen of the lysosome induces a conformational change that facilitates the interaction between NPC2 and the MLD of NPC1 and the transfer of cholesterol to the NTD [6]. It has been recently demonstrated that a loop–loop interaction between the CTD and NTD of NPC1 would keep the NTD in the proper orientation for receiving cholesterol from NPC2. Then, NPC2 changes its conformation to disassociate from the MLD-binding site [6], and the loop–loop interaction might be weakened to allow the NTD to deliver cholesterol to the SSD pocket in the membrane. How the NTD delivers cholesterol to the SSD is still not clear.

To date, 486 mutations in the *NPC1* gene have been reported worldwide (HGMD Professional database, Qiagen), and most of them are private or reported in a small number of families, with few exceptions. Indeed, the p.I1061T variant accounts for 20–25% of alleles in patients diagnosed in France [7] or the United Kingdom [8], but it seems to be much less frequent in countries from southern Europe [9,10]. The second most frequent mutation of *NPC1* in Europe is the p.P1007A, strongly associated with the variant biochemical phenotype [11,12]. Finally, the p.G992W mutation is highly frequent in Nova Scotian patients but rarely found in patients of other origins [11]. Only 27 mutations have been reported in the *NPC2* gene (HGMD Professional database, Qiagen), being the p.E20X the most frequently reported [1].

In 2009, the results of the molecular characterization of 44 Italian patients affected by NPC belonging to 35 unrelated families have been published [13]. A highly heterogeneous mutation profile of *NPC1* gene and a low frequency of the p.I1061T, similar to the frequency reported in Spain [9], were found. This suggests that there is a gradient of increasing frequency of the p.I1061T mutation from southeast to northwest Europe. Five alleles remained unidentified, suggesting the presence of deep intronic mutations or partial/total gene deletions, nonidentified by Sanger sequencing [13].

In the present multicentric study, we applied a laboratory diagnostic algorithm recently published [14] to complete the molecular analysis of *NPC1* gene in four patients previously reported and presenting pathogenic mutations in only one allele and to study 61 additional Italian NPC patients, providing an overall picture of the molecular characteristics of NPC patients diagnosed in Italy over the last 3 decades.

## 2. Materials and Methods

### 2.1. Patients

One hundred and five patients belonging to 83 unrelated families were included in a collaborative multicenter study aimed at characterizing the molecular bases of Niemann–Pick C in Italy. Patient’s phenotype classification was based on the age at onset of neurological symptoms as follows: severe infantile (SI, age at onset <2 years), late infantile (LI, age at onset 2–6 years), juvenile (J, age at onset 6–15 years), and adult (A, age at onset ≥15 years) [14]. Patients who died during the first month of life due to liver or respiratory insufficiency without signs of neurological involvement were classified as early infantile systemic lethal form (EISL). Asymptomatic patients and patients who did not present clinical or imaging signs of neurological involvement at last follow up were considered non-classifiable (NC).

The biochemical and molecular diagnosis of NPC was confirmed following the diagnostic algorithm recently published [14]. Four patients, previously reported as affected by NPC disease and presenting pathogenic mutations in only one allele [13], have been reanalyzed to complete the molecular diagnosis.

The research was performed in accordance with the declaration of Helsinki, and written consent was obtained from subjects or carers /guardians on behalf of the minors involved in the study.

### 2.2. Analysis of NPC1 and NPC2 Genes

Genomic DNA was extracted from peripheral blood leukocytes or fibroblasts using standard procedures. All *NPC1* and *NPC2* exons and their flanking regions were amplified by PCR [13]. Cycle sequencing was performed in the forward and reverse direction with the ABI PRISM Big Dye Terminator Cycle Sequencing Kit (Applied Biosystems, Warrington, UK) following the manufacturer’s instructions, and sequences were analyzed on the ABI Prism 3500 x l genetic analyzer (Applied Biosystems, Foster City, CA, USA). Putative mutations were confirmed by sequencing duplicate PCR products. Family studies, whenever possible, were also carried out.

The possible pathological nature of the novel *NPC1 or NPC2* sequence alterations detected was addressed by performing filipin staining of culture fibroblasts and western blot analysis or mRNA analysis of *NPC1* or *NPC2* whenever possible or by (i) screening 100 alleles from healthy control subjects for each alteration, (ii) querying SIFT [15] and Polyphen-2 [16] for predicting possible impact of an amino acid substitution on the structure and function of the protein, and (iii) consulting the predicted pathogenic effect reported by Adebali et al. [17]. A three-dimensional (3D) model of the wild-type NPC1 protein showing the localization of novel identified mutations, was built using Swiss Model Software (https://swissmodel.expasy.org/) employing the crystal structure PDB 5U73 as a template [18] The structure lacks the *N*-terminal domain and transmembrane helix 1.

### 2.3. Filipin Staining

Filipin staining was performed using the method described by Blanchette-Mackie et al. [19]. Briefly, the cells were rinsed with phosphate buffer saline (PBS) and fixed with 3% paraformaldehyde. After being washed with PBS, the cells were incubated with 1.5 mg of glycine/mL PBS for 10 min, stained with filipin (0.05 mg/mL, in PBS 10% FCS) for 2 hours, and examined using a Zeiss fluorescence microscope.

### 2.4. mRNA Analysis

Total RNA was isolated from cultured fibroblasts treated or not with anisomycin (100 μg/mL) for 4 hs using the QIA Shredder and the RNeasy Mini Kit (Qiagen GmbH, Hilden, Germany).

RT-PCR was performed as previously described using primers that allow the amplification of 10 overlapping fragments covering the entire NPC1 mRNA [20]. Each fragment was sequenced as described above.

### 2.5. MLPA Assay

The Multiplex Ligation-probe amplification (MLPA) reaction was performed to screen all exons of both *NPC1* and *NPC2* genes using SALSA MLPA probe set P183 (MRC-Holland, Amsterdam, the Netherlands) following the manufacturer’s instructions. Amplified products were separated using an ABI Prism 3500xl genetic analyzer (Applied Biosystems, Foster City, CA, USA), and the data were analyzed by Coffalyser Software (MRC-Holland, Amsterdam, the Netherlands).

### 2.6. Western Blot Analysis

20 μg of protein extracts were resolved on 4–20% gradient SDS PAGE gels and transferred to nitrocellulose membranes (Schleicher and Schuell, Keene, NH, USA). After overnight blocking with 5% nonfat dry milk in PBS-Tween 0.1% (PBS-T), the membranes were probed with anti NPC1 polyclonal antibody (Novus Biologicals, Littleton, USA; NB400-148) overnight at 4 °C. Anti-rabbit horse radish peroxidase (HRP) conjugated antibody was used as a secondary antibody. Immunoreactive bands were detected by enhanced chemioluminescence ECL (Amersham). The signals were normalized to those obtained for actin using a polyclonal anti-actin antibody (Sigma, St Louis, MO, USA; A2066).

### 2.7. Nomenclature

All mutations are described according to the recommended nomenclature [21,22]. Nucleotide numbers are derived from cDNA reference sequences: *NPC1*: NM_000271.4; *NPC2*: NM_006432.3.

## 3. Results

### 3.1. Distribution of NPC Clinical Phenotypes

Overall, we collected molecular data from 105 patients belonging to 83 unrelated families affected by NPC disease.

Seventy-seven families presented mutations in *NPC1* gene while six did in *NPC2*. Molecular, filipin staining, and clinical phenotype data are reported in Table 1.

**Figure 1:**
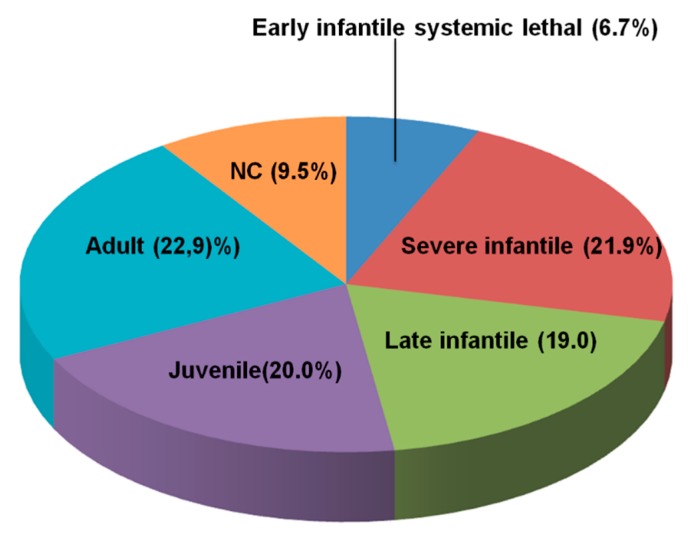
Relative distribution of the clinical phenotypes of Niemann–Pick type C1 (NPC1) patients based on the age at onset of first neurological signs. Severe infantile (SI): age at onset <2 years; Late infantile (LI): age at onset 2–6 years; Juvenile (J): age at onset 6–15 years; and Adult (A): age at onset ≥15 years. Patients who died during the first month of life due to liver or respiratory insufficiency without signs of neurological involvement were classified as early infantile systemic lethal form (EISL). NC: not classifiable.

The relative distribution of the clinical phenotypes based on the age at onset of first neurological signs is shown in Figure 1.

The adult form was the most frequent phenotype, while 10 patients remained unclassified, since at last follow up, they did not present signs or symptoms of neurological disease and MRI was normal in all cases. Among them, patients NP53, NP72, and NP73 (Table 1) were diagnosed due to neonatal cholestasis and remained free of neurological signs at the last follow up, at the age of 3, 0.5, and 1 years, respectively. Patients NP54 and NP56 presented with hepatosplenomegaly at 34 and 5 years of age, respectively. NP56 is currently 31 years old and presents only a mild splenomegaly. NP56sib is a younger brother of NP56. He is 18 years old and asymptomatic. Finally, patients NP82 and NP82sib are siblings of an NPC patient diagnosed on the bases of clinical signs and filipin staining who died at 6 years of age. Both NP82 and NP82sib are currently 37 and 40 years old, respectively. They are both asymptomatic.

### 3.2. NPC1 and NPC2 Mutational Profile

Among NPC1 families, the mutational spectrum was extremely heterogeneous. The complete genotype was established in 75 families, while in two (NP11 and NP23), the second allele was not identified (1.3% of alleles). In NP11, the mRNA was not available, and MLPA analysis was not performed; therefore, the presence of a deep intronic mutation or a large partial or total deletion of the allele could not be ruled out. Unexpectedly, even though a complete laboratory workup was performed in patient NP23, the second mutation remained unidentified. In this patient, the presence of a deep intronic mutation leading to the synthesis of an unstable transcript degraded by a mechanism different from the nonsense mediated decay (NMD) or the presence of a mutation in the promoter of the gene could be hypothesized. Patients NP13 and NP13sib had already been described in our previous work, and no expression of *NPC1* mRNA was detected. However, the mutation leading to the lack of NPC1 transcript was not identified at that time. Unfortunately, no samples were available to investigate the presence of deep intronic mutations leading to unstable transcripts rapidly degraded or large partial or complete deletions.

**Figure 2:**
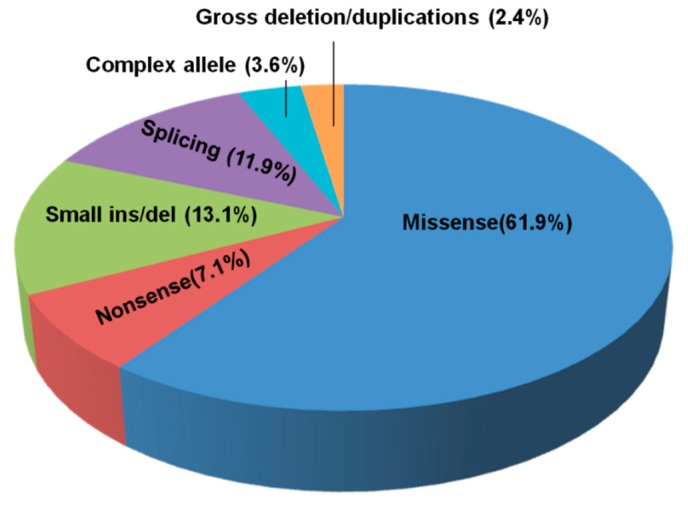
Spectrum of NPC1 type of mutation mutations.

Overall, 84 different *NPC1* alleles were identified: 52 were missense, 11 were small deletions/insertions, 10 were splicing mutations, 6 were nonsense variants, 3 were complex alleles, and 2 were gross deletions/duplications (Figure 2). Eighteen mutations were novel (Table 1—highlighted in red).

Fifty-seven mutations were private while only 27 variants were identified in more than one family (Table 2).

All *NPC1* identified variants presented a minor allele frequency (MAF) below 0.0001, except for the p.N222S, which has a MAF of 0.003 (http://gnomad.broadinstitute.org/about) [29]. As previously reported, the recurrent p.I1061T variant, which presents an allele frequency of 23% and 18% in patients from the United Kingdom [8] and France [7], respectively, accounted for only 3.9% of Italian alleles. A lower frequency was also reported in Spain [9], Greece [10], and the Czech Republic [30]. The most frequent *NPC1* mutation in our cohort was the p.F284Lfs*26 variant found in 5.8% of the alleles. This variant has been reported in the Greek population with a frequency of 3.8% [10]. The second most frequent mutation in our cohort was the p.P1007A, found in 5.2% of alleles. A similar frequency has been described in the Spanish population [9].

Data from eight patients belonging to six families affected by NPC2 have been collected. All of them carried missense or nonsense mutations at homozygous state (Table 1). Among the five different *NPC2* alleles, three were nonsense mutations. Interestingly, two nonrelated families presented the missense mutation p.M1L, both of them of Moroccan origin.

### 3.3. Filipin Staining

Filipin staining was available for 64 patients (59 NPC1 and 5 NPC2). All NPC2 patients presented with the classic biochemical phenotype, while 14/59 NPC1 patients presented the variant biochemical phenotype (Table 1, Supplementary Figure S1). As expected, 12 of them presented the adult form of the disease or were not classified since they are adults who did not display neurological disease at the last follow up.

Our data confirmed the association between the p.P1007A and p.G992W mutations and the variant biochemical phenotype [31]. Interestingly, all patients carrying the p.N222S storage low amounts of cholesterol within the lysosomes. The p.N222S variant was already reported in compound heterozygosity with the p.I1061T mutation in a single patient (35 years old) with adult-onset NPC with variant filipin staining [32].

### 3.4. Novel Mutations

Overall, 18 new variants were identified in the *NPC1* gene (Table 1 and Table 3). Three of them were considered pathogenetic, since they are gross deletions/duplications or nonsense variants (c.632_1553del; c.1554_1757dup and p.C227*). Among the remaining 15 variants, nine were missense, four were intronic variants, one was a complex allele, and one was an unidentified variant leading to a splicing defect. The pathogenic nature of 7/9 missense alleles was confirmed by means of filipin staining and western blot analysis of NPC1 protein in patients’ fibroblasts.

**Figure 3:**
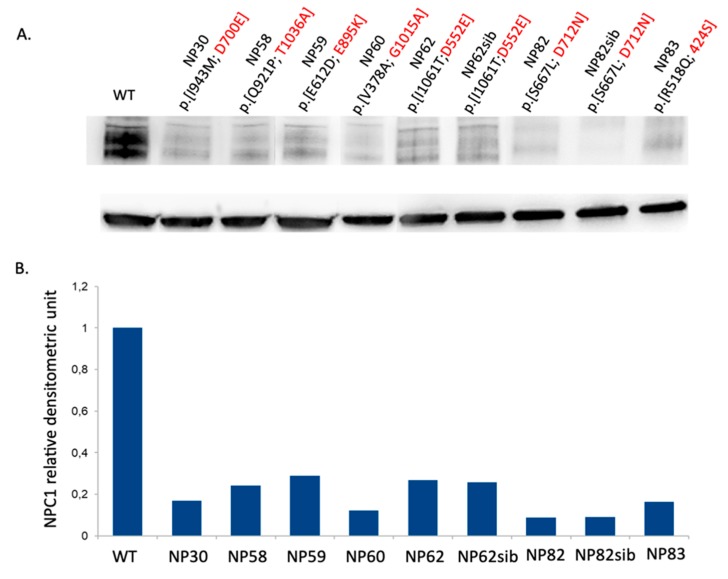
NPC1 protein abundance in patients carrying seven novel missense mutations. (**A**) Representative western blot analysis of NPC1 protein expression in NPC patients and normal control fibroblast cell lines. (**B**) The intensity of the NPC1 signals was normalized against actin. The NPC1 protein content in NPC fibroblasts was expressed as a percentage of the NPC1 protein content found in fibroblasts from a normal control.

As shown in Figure 3, cells from patients carrying mutations p.D700E, p.T1036A, p.D895K, p.G1015A, p.D712N, p.P424S, and p.D522E expressed extremely low amounts of NPC1 protein, suggesting that these mutations would lead to the synthesis of unfolded proteins that are rapidly degraded via endoplasmic-reticulum-associated degradation (ERAD). Unexpectedly, cells from siblings carrying the p.D522E mutation in compound heterozygosity with the p.I1061T variant (NP62 and NP62sib) expressed a very similar amount of NPC1 protein even though the filipin staining showed a classical and variant biochemical phenotype, respectively (Table 1 and Table 3).

**Figure 4:**
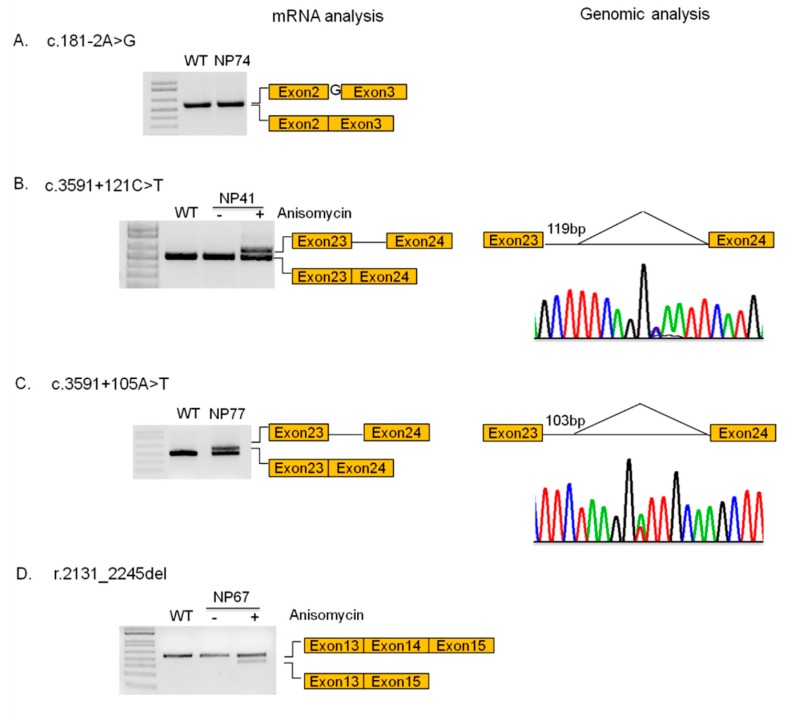
Functional analysis of four NPC1 splicing mutations. (**A**) The c.181-2A>G mutation caused the creation of a novel acceptor splice site leading to the insertion of a nucleotide in the mRNA transcript and the alteration of the reading frame; (**B**) the c.3591+121C>T and (**C**) the c.3591+105A>T mutations led to the generation of a cryptic acceptor splice site within intron 23, causing the retention of 119 and 103 nt, respectively, the shifting in the open reading frame, and the generation of a premature stop codon. The abnormal transcript resulting from the presence of c.3591+121C>T mutation was degraded via non-sense mediated decay (NMD). (**D**) In patient NP67, the skipping of exon 14 was observed (r.2131_2245del). The abnormal transcript was degraded via NMD.

The possible impact of 3/4 novel intronic variants on mRNA splicing was analyzed by studying the *NPC1* transcript expressed in patients’ fibroblasts treated or not with anisomycin, by RT-PCR and sequencing. The c.181-2A>G variant leads to the generation of a novel splicing acceptor site located 1 nucleotide (nt) upstream of the canonical 3’ss. As a consequence, the last nt of intron 2 is retained within the mature transcript causing a frameshift of the open reading frame and the generation of a premature stop codon that would eventually result in the synthesis of a truncated protein (p.E61Gfs*24) (Figure 4A).

The *NPC1* mRNA analysis in fibroblasts from patients NP41 and NP77 showed the partial retention of 119 and 103 nt, respectively, of intron 23 within the mature transcripts with the consequent shifting in the open reading frame and the generation of a premature stop codon. In the case of patient NP41, the aberrant transcript is degraded via NMD, while in the case of the NP77 patients, the aberrant transcript would eventually be translated in a truncated protein (p.F1199Sfs*21). Sequencing analysis of intron 23 demonstrated the presence of c.3591+121C>T and c.3591+105A>T variants in patients NP41 and NP77, respectively. Both variants led to the generation of a cryptic acceptor splice site within intron 23 (Figure 4B,C).

Finally, cells from patient NP67 showed the presence of an aberrant transcript in which the exon 14 was completely excluded (r.2131_2245del) leading once again, to the shifting of the open reading frame and the generation of a premature stop codon. However, this transcript was only present in cells treated with anisomycin, indicating that it is degraded via NMD (Figure 4D). Unfortunately, we were unable to identify the genomic mutation responsible for this splicing defect. However, the analysis of untreated cells showed the presence of normal spliced mRNA transcribed from the unidentified allele, indicating that the unidentified mutation would not completely abrogate normal splicing. Interestingly, this patient displayed a variant biochemical phenotype and was affected by the adult clinical phenotype, suggesting that the amount of normal *NPC1* transcript generated from this allele would be enough to promote a partial clearance of lysosomal cholesterol and prevent the development of a severe form of NPC.

The possible pathogenic nature of missense variants was also analyzed in silico. As shown in Table 3, the results obtained in vitro were supported by at least one of the prediction programs except for mutations p.D552E and p.E895K, which were predicted as benign. Unfortunately, cells from patients carrying new mutations p.R997S; p. [C160R; N222S]; p.S1148I and c.1654+6delTA were not available. The possible effect of these variants on NPC1 protein or *NPC1* mRNA splicing has been analyzed only using bioinformatic tools. As shown in Table 3, mutations p.C160R and p.S1148I were predicted to be deleterious while the p.R997S variant was predicted to be tolerated. Finally, the c.1654+6delTA variant would not affect the splicing process since no changes in the strength of the donor splicing site was predicted by NN splice (data not shown).

### 3.5. Novel NPC1 Mutations and Protein Structure

**Figure 5:**
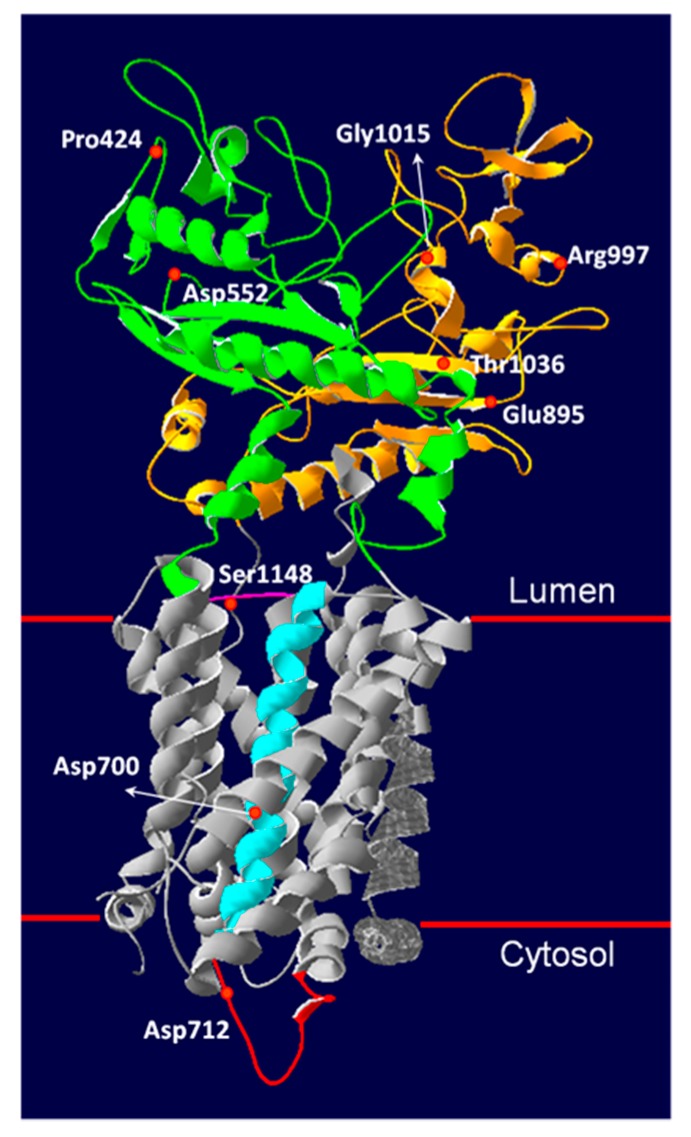
NPC1 protein structure and variants. The structure of NPC1 (PDB 5U73) showed the location of the NPC1 variants identified in this study. Functional domains containing NPC1 variants are indicated in different colors. MLD, middle luminal domain = green; CTD, C-terminal domain = yellow; TM5 = blue; Cytosolic loop = red; lumenal loop between TM5 and TM6 = pink.

Since the protein structure of NPC1 is available [6,18], we tried to gain insights into the possible effect of novel missense variants on NPC1 protein structure (Figure 5). The NPC1 protein has 13 transmembrane helices (TM) and three lumen-exposed domains. The N-terminal lumenal domain (NTD) which contains a cholesterol-binding site; the middle lumenal domain (MLD), which binds the NPC2 protein, presumably to assist in the transfer of cholesterol from NPC2 to NPC1 protein; and the C-terminal domain (CTD), which interacts with the NTD, contributing to keeping its proper orientation for receiving cholesterol from NPC2.

Two mutations (p.P424S and p.D552E) were reported in the MLD. The Pro424 residue is involved in the molecular ligand recognition [6], and therefore the substitution of proline with serine would affect the interaction between MTD and NPC2 protein. The Asp552 is a surface residue in the MLD without interaction with the neighboring residues. Thus, substitution of this residue with glutamate, an amino acid characterized by similar properties, seems to have no significant structural impact, as predicted by in silico programs. However, in vitro studies demonstrated that the siblings carrying the p.D552E present a very low expression of NPC1 protein and a similar clinical phenotype but a different pattern of cholesterol accumulation (Table 1 and Table 3). Based on these results, it is possible to speculate that the p.D552E variant is a mild mutation associated with the adult clinical phenotype and moderate cholesterol storage, while other factors might modify the extent of cholesterol accumulation.

Four mutations (p.E895L; p.R997S; p.G1015A and p.T1036A) occurred in the CTD, where the majority of NPC-disease-causing mutations have been identified. The Glu895 residue forms an intrahydrogen bond with the Tyr394 located in the MLD. Likely, the substitution of Glu895 with Leucine might disrupt the hydrogen bond interaction, therefore altering the MLD–CTD 3D conformation. This would be in agreement with the in vitro studies showing a very low expression of NPC1 protein in the patient carrying this variant and a massive accumulation of unesterified cholesterol within the lysosomes (classic biochemical phenotype). The Arg997 residue interacts with Asp964, Gly993, and Asp994 residues within the same domain. The substitution with serine might impair the interaction with Asp964, compromising the CTD stability. However, this variant was predicted as benign by all in silico tools and no cells were available for in vitro studies. The p.G1015A determines the substitution of a Glycine with an Alanine. The Gly1015 residue is highly conserved among species, and a pathogenic mutation involving the same amino acid residue, p.G1015V, has been previously identified in a patient characterized by juvenile NPC disease with psychomotoric retardation and early death [33]. The Thr1036 seems to stabilize two β strands and the surrounded α-helices in CTD. The substitution with Alanine might disrupt the 3D CTD conformation. Substitutions with either Methionine or Lysine have been reported [8,34], and the corresponding mutations p.T1036M and p.T1036K have been associated with an infantile and adult clinical phenotype, respectively. The p.D700 residue is located in the sterol sensing domain (SSD) constituted by TM3-7. The SSD is a highly conserved motif in several proteins involved in cholesterol sterol homeostasis. In particular, the SSD of NPC1 directly binds cholesterol, which is critical for the transport function of NPC1 [35]. The p.D700E determines the substitution of aspartate with glutamate. A pathogenic mutation, p.D700N, has been previously reported [36] in a patient characterized by a severe clinical phenotype. The Asp712 residue is located on the cytosolic side of the TM5. Its substitution with the positively charged Asparagine would likely lead to a conformational change of the cytosolic loop. A similar effect might be observed in the presence of p.S1148I mutation, which determines the substitution of serine, a small hydrophilic amino acid, with isoleucine, a large hydrophobic amino acid in the lumenal domain between TM10 and TM11.

### 3.6. Genotype–Phenotype Correlation

**Figure 6:**
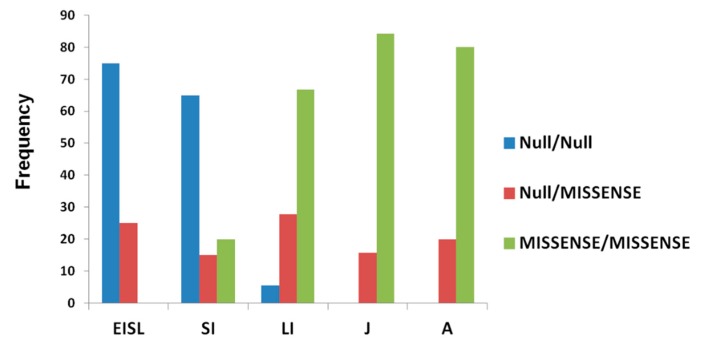
Distribution of genotypes among NPC phenotypes. Genotypes were categorized as homozygote (null/null) or compound heterozygote (null/missense) for severe mutations and homozygote for a missense mutation (missense/missense). 75% and 65% of EISL (early infantile systemic lethal) and SI (severe infantile) patients, respectively, were homozygote for severe mutations, while 66.7% of LI (Late infantile) 84.2% of J (juvenile) , and 80% of A (adult) patients presented missense mutations in both alleles. No homozygote or compound heterozygote for severe mutations was detected in J or A patients.

Genotype–phenotype correlations are difficult to establish in patients affected by NPC1, mainly due to the private nature of most *NPC1* mutations. Although our data confirm the wide phenotypic variability among patients carrying the same mutations, even within the same family, some general observations can be made from data obtained in our cohort. As shown in Table 1 and Figure 6, the vast majority of EISL and SI patients presented mutations that can be classified as very severe (null mutations) since they are partial deletions, frameshift/nonsense mutations, or splicing mutation proven to be severe by functional analysis in both alleles (75% and 65% of EISL and SI patients, respectively), while most LI, J, and A patients presented missense mutations in both alleles (66.7%, 84.2%, and 80% of LI, J, and A patients, respectively). None of the J or A patients were homozygous or compound heterozygous for severe mutations. Furthermore, in the adult group, only four patients presented severe mutations and always in compound heterozygosity with missense mutations already suggested to be associated with the late-onset phenotype (p.R978C and p.G992R) [37].

The pathogenic nature of the p.N222S mutation has been controversial. In this study, we have identified three patients who presented this mutation in compound heterozygosity. One of them remained unclassified since she is 40 years old and so far, did not develop neurological disease, and one presented the neurological adult phenotype. These results are consistent with data reporting this mutation in visceral-only or adult-onset NPC1 cases [2]. However, patient NP71 developed neurological symptoms at the age of 2 years. Although the presence of an additional variant in cis with the p.N222S was excluded by MLPA and mRNA analysis, the presence of mutations in other genes that would modify the clinical expression of the disease cannot be ruled out.

As stated above, the presence of this variant was associated with the variant biochemical phenotype in all patients for whom the filipin staining was available.

It is worth noting that the p.V378A was identified in two patients affected by the adult form of the disease. So far, this mutation has been reported in only one juvenile patient and in four other adults [8,31,38], strongly suggesting that it would be associated with the late-onset phenotype. The p.P474L mutation, found in two juvenile patients, seems to correlate with the juvenile phenotype since it has been reported in other patients affected by this form of the disease [20,30,39,40].

All studied NPC2 patients were homozygous, and a good genotype/phenotype correlation was found. Indeed, 6/8 patients belonging to four families carried severe variants; four harbored nonsense mutations, and two carried a mutation that affects the translation start codon probably leading to the complete absence of NPC2 protein expression. All these patients presented a severe phenotype (EISL or EI). Conversely, two siblings presented the p.L9P mutation, located within the signal peptide; both were affected by the adult clinical phenotype.

## 4. Conclusions

This paper provides an overview of the molecular genetics of Niemann–Pick type C disease in Italy and several conclusions can be drawn from the analysis of the obtained results.

First, we have shown that the adult phenotype was the most frequently identified among patients affected by NPC disease in Italy, accounting for 22.9% of patients. This data is in contrast with the distribution of clinical phenotypes previously published by Fancello et al. [13]. Indeed, in the previously studied Italian cohort, only 12% of patients presented the adult form of NPC while 32% of patients were affected by the late infantile phenotype. This discrepancy is not surprising since during the last decade, the increased awareness of NPC disease among the medical community and within the families of affected individuals, as well as the improvement of diagnostic tools, has facilitated the diagnosis of patients affected by milder and atypical phenotypes.

Second, the application of the diagnostic algorithm recently published [14] led to the identification of 98.8% of *NPC* alleles. This result highlights the need to perform an in-depth molecular analysis of *NPC1* and *NPC2* genes to establish the complete genotype. Indeed, six alleles (3.6%) displayed deep intronic variants or partial deletions/duplications that cannot be detected by sequencing analysis.

Third, this study confirmed the highly heterogeneous profile of mutations in patients affected by NPC1, and the low frequency of the common p.I1061T mutation among the Italian cohort. Due to this broad genotypic variability, it is difficult to correlate the genotype and the phenotype. However, the early infantile severe lethal phenotype and the severe infantile phenotype were mainly associated with the presence of null mutations in both alleles, while all patients affected by the juvenile and adult phenotypes carried missense mutations at least in one allele. Furthermore, 84.2% and 80%, respectively, carried this type of mutation in both alleles. Besides these general considerations, it is worth noting that, as already reported [41,42], a high phenotypic variability was observed even within the same family, suggesting that additional genetic and/or nongenetic factors might act as disease modifiers. Indeed, the influence of the genetic background on the disease onset, severity, and survival has been extensively studied in different NPC1 mouse models [43,44,45,46,47]. The possible influence of nongenetic factors on the phenotypic expression of NPC disease and genetic diseases in general, has been poorly explored. However, the development of divergent phenotypes over the years in monocygote twins, due to an increasing variability in epigenetic events which are sensitive to external stimuli, such as DNA methylation and histone acetylation has been reported, bridging the gap between environmental and genetic factors [48,49].

Fourth, 12/14 patients displaying a variant biochemical phenotype present a less severe form of the disease. Although the pathogenic nature of the p.N222S mutation is controversial, we found 3 patients who presented this variant in compound heterozygosity; all of them displayed a variant biochemical phenotype. Our data are in line with those of Wassif et al. [2] suggesting that this variant might be associated with the visceral-only or adult-onset NPC1 cases.

Fifth, we described 18 novel *NPC1* variants, and the pathogenic nature of 11 of them was established by means of mRNA or protein expression and cholesterol accumulation studies.

Finally, we reported the molecular data of seven patients belonging to five families presenting mutations in the *NPC2* gene. Five of these patients presented a severe phenotype. Conversely to patients with mutations in *NPC1*, all NPC2 patients were homozygous and a very good genotype/phenotype correlation was found.

## Figures and Tables

**Table 1:** Genotype and clinical and biochemical phenotype of Niemann-Pick C patients analyzed in this study.

Patient Code	Allele1	Allele 2	Clinical Phenotype	Filipin	Gene	Reference
NP3 sib	c.852delT (p.F284Lfs*26)	c.852delT (p.F284Lfs*26)	EISL	NA	NPC1	[13]
NP10 sib	c.1819C>T (p.R607*)	c.3614C>A (p.T1205K)	EISL	NA	NPC1	[13]
NP18 sib	c.2800C>T (p.R934*)	c.2872C>T (R958*)	EISL	NA	NPC1	[13]
NP35	c.1029dupG (p.S344Vfs*36)	c.1029dupG (p.S344Vfs*36)	EISL	NA	NPC1	[23]
NP20	c.1A>T (p.M1L)	c.1A>T (p.M1L)	EISL	CLASSIC	NPC2	[13]
NP78	c.133C>T (p.Q45*)	c.133C>T (p.Q45*)	EISL	CLASSIC	NPC2	[24]
NP36	c.436C>T (P.Q146*)	c.436C>T (Q146*)	EISL	CLASSIC	NPC2	[12]
NP3	c.852delT (p.F284Lfs*26)	c.852delT (p.F284Lfs*26)	EI	NA	NPC1	[13]
NP7	c.2972_2973delAG (p.Q991Rfs*15)	c.2972_2973delAG (p.Q991Rfs*15)	EI	CLASSIC	NPC1	[13]
NP9	c.1901A>G (p.Y634C)	c.3562delG (p.E1188Kfs*54)	EI	CLASSIC	NPC1	[13]
NP10	c.1819C>T (p.R607*)	c.3614C>A (p.T1205K)	EI	CLASSIC	NPC1	[13]
NP12	c.93_94delTG (p.C31Wfs*26)	c.93_94delTG (p.C31Wfs*26)	EI	NA	NPC1	[13]
NP13	r.0	r.0	EI	NA	NPC1	[13]
NP13sib	r.0	r.0	EI	NA	NPC1	[13]
NP14	c.3467A>G (p.N1156S)	c.3467A>G (p.N1156S)	EI	NA	NPC1	[13]
NP16	c. 3613-3614insA (p.T1205Nfs*53)	c. 3613-3614insA (p.T1205Nfs*53)	EI	CLASSIC	NPC1	[13]
NP18	c.2800C>T (p.R934*)	c.2872C>T (p.R958*)	EI	CLASSIC	NPC1	[13]
NP22	c.2762A>C (p.Q921P)	c.2339T>G (p.V780G)	EI	CLASSIC	NPC1	[13]
NP37	c.852delT (p.F284Lfs*26)	c.852delT (p.F284Lfs*26)	EI	CLASSIC	NPC1	[25]
NP38	c.2800C>T (p.R934*)	c.3235T>C (p.F1079L)	EI	CLASSIC	NPC1	[23]
NP39	c.2761C>T (p.Q921*)	c.2761C>T (p.Q921*)	EI	NA	NPC1	[23]
NP40	c.852delT (p.F284Lfs*26)	c.852delT (p.F284Lfs*26)	EI	CLASSIC	NPC1	[26]
NP71	c.665A>G (p.N222S)	c.2861C>T (p.S954L)	EI	VARIANT	NPC1	This study
NP74	c.181-2A>G (p.E61Gfs*24)	c.1553+1G>A (p.R518Gfs*7)	EI	CLASSIC	NPC1	This study
NP75	c.3322dupG (p.A1108Gfs*13)	c.3322dupG (p.A1108Gfs*13)	EI	CLASSIC	NPC1	[24]
NP76	c.2819C>T (p.S940L)	c.2819C>T (p.S940L)	EI	NA	NPC1	This study
NP82	**c.1554_1757dup (p.A519_E586dup)**	**c.1554_1757dup (p.A519_E586dup)**	EI	CLASSIC	NPC1	This study
NP34	c.58G>T (p.E20*)	c.58G>T (p.E20*)	EI	NA	NPC2	[13]
NP34Sib	c.58G>T (p.E20*)	c.58G>T (p.E20*)	EI	NA	NPC2	[13]
NP80	c.1A>T (p.M1L)	c.1A>T (p.M1L)	EI	CLASSIC	NPC2	This study
NP1	c.2776G>A (p.A926T)	c.3731 T>C (p.L1244P)	LI	CLASSIC	NPC1	[13]
NP2	c.3571C>T (p.L1191F)	c.3571C>T (p.L1191F)	LI	CLASSIC	NPC1	[13]
NP4	c.3068T>G (p.V1023G)	c.3068T>G (p.V1023G)	LI	NA	NPC1	[13]
NP5	c.1535A>G (p.H512R)	c.3056A>G (p.Y1019C)	LI	CLASSIC	NPC1	[13]
NP6	c.852delT (p.F284Lfs*26)	c.3056A>G (p.Y1019C)	LI	NA	NPC1	[13]
NP11	c.2972_2973delAG (p.Q991Rfs*15)	?	LI	VARIANT	NPC1	[13]
NP15	c.852delT (p.F284Lfs*26)	c.852delT (p.F284Lfs*26)	LI	CLASSIC	NPC1	[13]
NP17	c.710C>T (p.P237L)	c.3304C>T (p.L1102F)	LI	NA	NPC1	[13]
NP19	c.3019C>G (p.P1007A)	c.3614C>A (p.T1205K)	LI	NA	NPC1	[13]
NP25	c. 3182 T>C (p.I1061T)	c. 3182 T>C (p.I1061T)	LI	CLASSIC	NPC1	[13]
NP26	c. 298T>A (p.C100S)	c.[3424T>C ; 1943T>A] (p.[L648H; M1142T])	LI	CLASSIC	NPC1	[13]
NP27	c.2662C>T (p.P888S)	c.2761C>T (p.Q921*)	LI	NA	NPC1	[13]
NP28	c.2762A>C (p.Q921P)	c. 3182 T>C (p.I1061T)	LI	CLASSIC	NPC1	[13]
NP30	c.2829C>G (p.I943M)	**c.2100C>G (p.D700E)**	LI	CLASSIC	NPC1	[25]
NP41	c.3493G>A (p.V1165M)	**c.3591+121C>T (p.0)**	LI	CLASSIC	NPC1	[26]
NP42	c.3019 C>G (p.P1007A)	c.3734_3735delCT (p.P1245Rfs12*)	LI	CLASSIC	NPC1	[26]
NP43	c.3493G>A (p.V1165M)	c.58-3T>G (p.?)	LI	CLASSIC	NPC1	[26]
NP61	c.3614C>A (p.T1205K)	c.3614C>A (p.T1205K)	LI	NA	NPC1	This study
NP81	c.681T>A (p.C227*)	c.2861C>T (p.S954L)	LI	NA	NPC1	This study
NP58	**c.3106A>G (p.T1036A)**	c.2762A>G (p.Q921P)	J	CLASSIC	NPC1	This study
NP19sib	c.3019 C>G (p.P1007A)	c.3614C>A (p.T1205K)	J	NA	NPC1	[13]
NP24	c.2800C>T (p.R934*)	c.2292G>A (p.A750_G765del)	J	CLASSIC	NPC1	[13]
NP24sib	c.2800C>T (p.R934*)	c.2292G>A (p.A750_G765del)	J	CLASSIC	NPC1	[13]
NP25Sib	c. 3182 T>C (p.I1061T)	c.3182 T>C (p.I1061T)	J	CLASSIC	NPC1	[13]
NP29	c.3056A>G (p.Y1019C)	c.3056A>G (p.Y1019C)	J	CLASSIC	NPC1	[13]
NP32Sib	c.2762A>C (p.Q921P)	c.2903A>G (p.N968S)	J	NA	NPC1	[13]
NP32Sib^1^	c.2762A>C (p.Q921P)	c.2903A>G (p.N968S)	J	NA	NPC1	[13]
NP43sib	c.3493G>A (p.V1165M)	c.58-3T>G (p.?)	J	NA	NPC1	[26]
NP44	c.2662C>T (p.P888S)	c.2662C>T (p.P888S)	J	CLASSIC	NPC1	[23]
NP44sib	c.2662C>T (p.P888S)	c.2662C>T (p.P888S)	J	NA	NPC1	[23]
NP45	c.3467A>G (p.N1156S)	c.3467A>G (p.N1156S)	J	NA	NPC1	[23]
NP46	c.1501G>T (p.D501Y)	c.1421 C>T (p.P474L)	J	CLASSIC	NPC1	[23]
NP47	c.2819C>T (p.S940L)	c.2291C>T (p.A764V)	J	CLASSIC	NPC1	[23]
NP56sib^1^	c.2974G>T (p.G992W)	c.1351G>A (p.E451K)	J	VARIANT	NPC1	[23]
NP57	c.3493G>A (p.V1165M)	c.2795+1G>C (p.0)	J	CLASSIC	NPC1	[27]
NP63	c.1421C>T (p.P474L)	c.1421C>T (p.P474L)	J	CLASSIC	NPC1	[25]
NP59	**c.2683G>A (p.E895K)**	c.1836A>C (p.E612D)	J	CLASSIC	NPC1	This study
NP70	c.352_353delAG (p.Q119FS*8)	c.2861C>T (p.S954L)	J	NA	NPC1	This study
NP77	c.3591+105A>T (p.F1199Sfs*21)	c.3467A>G (p.N1156S)	J	CLASSIC	NPC1	This study
NP79	**c.3443G>T (p.S1148I)**	c.3019C>G (p.P1007A)	J	NA	NPC1	This study
NP84	**c.1270C>T (p.P424S)**	c.1553G>A (p.R518Q)	A	CLASSIC	NPC1	This study
NP21	c.1415T>A (p.L472H)	c.3230G>A (p.R1077Q)	A	CLASSIC	NPC1	[13]
NP23	c.1907C>T (p.S636F)	c.(?)	A	CLASSIC	NPC1	[13]
NP31	c.665A>G (p.N222S)	c.3560C>T (p.A1187V)	A	VARIANT	NPC1	[13]
NP33	c.1166G>T (p.R389L)	c.1166G>T (p.R389L)	A	NA	NPC1	[13]
NP48	c.882-28A>G (p.0)	c.2932C>T (p.R978C)	A	VARIANT	NPC1	[28]
NP48sib	c.882-28A>G (p.0)	c.2932C>T (p.R978C)	A	NA	NPC1	[28]
NP50	c.3493G>A (p.V1165M)	c.3019C>G (p.P1007A)	A	VARIANT	NPC1	[25]
NP51	c.2974G>C (p.G992R)	c. 2130dupG (p.R711Efs*3)	A	CLASSIC	NPC1	[23]
NP51Sib	c.2974G>C (p.G992R)	c. 2130dupG (p.R711Efs*3)	A	NA	NPC1	[23]
NP52	c.3019C>G (p.P1007A)	c.3019C>G (p.P1007A)	A	NA	NPC1	[23]
NP56sib^2^	c.2974G>T (p.G992W)	c.1351G>A (p.E451K)	A	VARIANT	NPC1	[23]
NP56sib^3^	c.2974G>T (p.G992W)	c.1351G>A (p.E451K)	A	VARIANT	NPC1	[23]
NP60	c.1133T>C (p.V378A)	**c.3044G>C (p.G1015A)**	A	CLASSIC	NPC1	This study
NP60sib	c.1133T>C (p.V378A)	**c.3044G>C (p.G1015A)**	A	NA	NPC1	This study
NP62	**c.1656T>A (p.D552E)**	c.3182T>C (p.I1061T)	A	CLASSIC	NPC1	This study
NP62sib	**c.1656T>A (p.D552E)**	c.3182T>C (p.I1061T)	A	VARIANT	NPC1	This study
NP64	c.632_1553del (p.D211Gfs*6)	**c.1654+6delTA (p.?)**	A	NA	NPC1	This study
NP65	c.3493G>A (p.V1165M)	c.3493G>A (p.V1165M)	A	NA	NPC1	This study
NP66	c.3019C>G (p.P1007A)	c.3019C>G (p.P1007A)	A	NA	NPC1	This study
NP67	c.2728G>A (p.G910S)	**r.2131_2245del (p.0; p.=)**	A	VARIANT	NPC1	This study
NP68	c.1907C>T (p.S636F)* c.2932C>T (p.R978C) c.3467A>G (p.N1156S)		A	NA	NPC1	This study
NP69	**c.2991A>T (p.R997S)**	c.3493G>A (p.V1165M)	A	NA	NPC1	This study
NP49	c.26T>C (p.L9P)	c.26T>C (p.L9P)	A	CLASSIC	NPC2	[25]
NP49sib	c.26T>C (p.L9P)	c.26T>C (p.L9P)	A	NA	NPC2	This study
NP8	c.1339C>T (p.Q447*)	c.1339C>T (p.Q447*)	NC	CLASSIC	NPC1	[13]
NP29	c.3056A>G (p.Y1019C)	c.3056A>G (p.Y1019C)	NC	CLASSIC	NPC1	[13]
NP53	c.2972_2973delAG (p.Q991Rfs*15)	c. 3182 T>C (p.I1061T)	NC	CLASSIC	NPC1	[23]
NP54	c.2689C>A (p.H897N)	c.665A>G (p.N222S)	NC	VARIANT	NPC1	[23]
NP56	c.2974G>T (p.G992W)	c.1351G>A (p.E451K)	NC	VARIANT	NPC1	[23]
NP56sib	c.2974G>T (p.G992W)	c.1351G>A (p.E451K)	NC	VARIANT	NPC1	[23]
NP72	c. 3613-3614insA (p.T1205Nfs*53)	c. 3182 T>C (p.I1061T)	NC	NA	NPC1	This study
NP73	**c.[478T>C; c.665A>G] (p.[C160R; N222S])**	**c.3106A>G (p.T1036A)**	NC	NA	NPC1	This study
NP83	**c.2134G>A (p.D712N)**	c.2000C>T (p.S667L)	NC	VARIANT	NPC1	This study
NP83 sib	**c.2134G>A (p.D712N)**	c.2000C>T (p.S667L)	NC	VARIANT	NPC1	This study

SI = severe infantile; LI = late infantile; J = juvenile; A = adult; EISL = early infantile systemic lethal form; NC = non-classifiable; NA = not available. Novel mutations are indicated in red. RefSeq NPC1 and NPC2 cDNA NM_000271.5 and NM_006432.5. For cDNA numbering, +1 corresponds to the A of the first ATG translation initiation codon. RefSeq NPC1 and NPC2 protein NP_000262.2 and NP_006423.1. * Segregation analysis was not possible in this patient. Patients with the same NP number are members of the same family. Siblings within a family were identified with the same NP number followed by sib; sib^1^; sib^2^, sib^3^).

**Table 2:** Frequency of NPC1 mutant alleles identified in more than one allele.

NPC1 Variant	*n* of Alleles	Frequency (%)
p.F284Lfs*26	9	5.8
p.P1007A	8	5.2
p.V1165M	7	4.5
p.N1156S	6	3.9
p.I1061T	6	3.9
p.Q991Rfs*15	4	2.6
p.T1205K	4	2.6
p.Y1019C	4	2.6
p.Q921P	4	2.6
p.S954L	3	1.95
p.R934*	3	1.95
p.Q921*	3	1.95
p.P888S	3	1.95
p.P474L	3	1.95
p.S940L	3	1.95
p.N222S	3	1.95
p.S334Vfs*36	2	1.3
p.C31Wfs*26	2	1.3
p.T1205Nfs*53	2	1.3
p.L1191F	2	1.3
p.V1023G	2	1.3
p.S636F	2	1.3
p.R389L	2	1.3
p.R978C	2	1.3
p.Q447*	2	1.3
p.A1108Gfs*13	2	1.3
p.T1036A	2	1.3

**Table 3:** Clinical, biochemical, and molecular characteristics of the identified NPC1 novel mutations.

Patient Code	Nucleotide Substitution	Predicted Aminoacid Change	Filipin Staining	SIFT	Polyphen	Classification Following Adebali et al. [17]
NP74	c.181-2A>G	p.E61Gfs*24	CLASSIC	NA	NA	NA
NP73	c. [478T>C; c.665A>G]	p [C160R; N222S]	NA			NA
NP64	c.632_1553del	p.D211Gfs*6	NA	NA	NA	NA
NP81	c.681T>A	p.C227*	NA	NA	NA	NA
NP84	c.1270C>T	p.P424S	CLASSIC	Benign	Benign	Damaging
NP82	c.1554_1757dup	p.A519_E586dup	CLASSIC	NA	NA	NA
NP64	c.1654+6delTA	p.?	NA	NA	NA	NA
NP62 NP62sib	c.1656T>A	p.D552E	CLASSIC VARIANT	Benign	Benign	Benign
NP30	c.2100C>G	p.D700E	CLASSIC	Damaging	Damaging	Damaging
NP83 NP83sib	c.2134G>A	p.D712N	VARIANT	Damaging	Damaging	Damaging
NP59	c.2683G>A	p.E895K	CLASSIC	Benign	Benign	Benign
NP69	c.2991A>T	p.R997S	NA	Tolerated	Benign	Benign
NP60 NP60sib	c.3044G>C	p.G1015A	CLASSIC	Damaging	Damaging	Damaging
NP58 ^#^	c.3106A>G	p.T1036A	CLASSIC	Damaging	Damaging	Damaging
NP79	c.3443G>T	p.S1148I	NA	Damaging	Probably damaging	Damaging
NP77	c.3591+105A>T	p.F1199Sfs*21	CLASSIC	NA	NA	NA
NP41	c.3591+121C>T	p = 0	CLASSIC	NA	NA	NA
NP67	r.2131_2245del	(p.0; p.=)	VARIANT	NA	NA	NA

^#^ The same mutation has been found in patient NP73 who presented with neonatal cholestasis who remained free of neurological signs at last follow up (age 1 year old). NA: not available.

## Supplementary Materials

The following are available online at https://www.mdpi.com/2077-0383/9/3/679/s1, Figure S1: Representative filipin staining of human skin fibroblasts of a normal control and Niemann-Pick patients.
