# Supplementary material for: Molecular Genetics of Niemann–Pick Type C Disease in Italy: An Update on 105 Patients and Description of 18 NPC1 Novel Variants
Source: J Clin Med. 2020 Mar 3;9(3):679. doi: 10.3390/jcm9030679 (PMC7141276; doi:10.3390/jcm9030679)
Supplement: Supplementary file 1 [file jcm-09-00679-s001.pdf]

Supplementary Figure 1

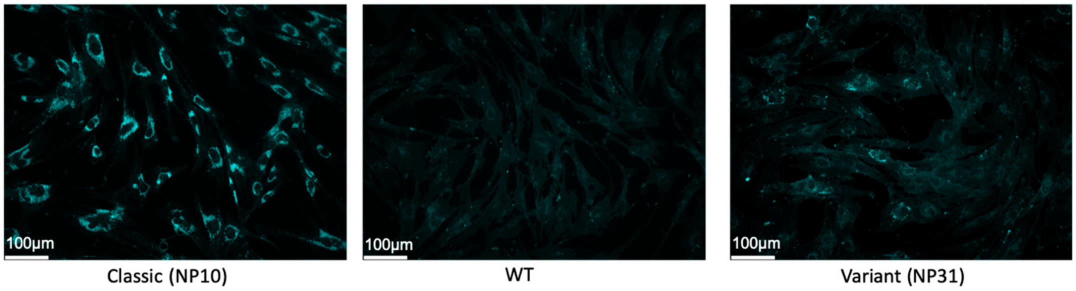

Figure S1. Representative filipin staining of human skin fibroblasts of a normal control and patients showing a classic (NP10) and variant (NP31) pattern of unesterified cholesterol accumulation within the lysosomes.
